# Karyotype characterization and cytogenetic diversity of *Medicago sativa* L. (Leguminosae) populations from the Lake Region of Türkiye

**DOI:** 10.3897/compcytogen.20.205237

**Published:** 2026-09-16

**Authors:** Derya Güloğlu

**Affiliations:** 1 Department of Plant and Animal Production, Isparta University of Applied Sciences, Atabey, 32200 Isparta, Türkiye Department of Plant and Animal Production, Isparta University of Applied Sciences Isparta Türkiye

**Keywords:** Autotetraploidy, B-chromosomes, cytogenetics, heteromorphic chromosomes, karyotype asymmetry

## Abstract

*Medicago sativa* Linnaeus, 1753 (Leguminosae) is a globally important autotetraploid forage crop with a complex genome. Local populations adapted to specific ecological niches, such as those in Türkiye’s Lake Region, represent valuable genetic resources for breeding. However, their cytogenetic diversity remains poorly characterized. This study aimed to perform detailed karyotype analyses of eight alfalfa populations from this region to assess chromosome number, morphology, asymmetry, and structural variants. Root tips from eight populations collected from Afyonkarahisar, Burdur, Isparta, and Konya provinces were analyzed using aceto-iron-hematoxylin staining and the squash technique. Chromosome measurements, arm ratios, centromeric indices, karyotype formulas, and asymmetry indices (intrachromosomal A1 and interchromosomal A2) were determined. Descriptive parameters (TF%, DRL, DI) and clustering analyses were used to compare cytogenetic diversity among populations. In accordance with current karyomorphological recommendations, mean centromeric asymmetry (MCA), coefficient of variation of chromosome length (CVCL), and coefficient of variation of centromeric index (CVCI) were additionally reconstructed from the chromosome-pair mean measurements reported in the paper. All populations were confirmed as autotetraploid (2n = 4x = 32). Karyotypes consisted predominantly of metacentric chromosomes, with fewer submetacentrics, and all possessed four satellite chromosomes. Reconstructed MCA ranged from 11.86% (Bolvadin-3) to 15.36% (Yalvaç-3), CVCL ranged from 8.81% (Akşehir-2) to 12.69% (Bolvadin-3), and CVCI ranged from 7.77% (Bucak-2) to 11.63% (Yalvaç-3). A1 ranged from 0.208 (Bolvadin-3) to 0.258 (Hüyük-2), and A2 ranged from 0.096 (Yeşilova-3) to 0.149 (Hüyük-2). B-chromosomes (2n = 32 + 2B) were detected exclusively in Bucak-2, and heteromorphic chromosome pairs were observed in Hüyük-2 and Uluborlu-1. Hüyük-2 remained the most structurally distinctive population because of its high A1 and A2 values, heteromorphic chromosome pair, enlarged satellite, and Stebbins 2C classification. Cluster analysis based on asymmetry indices separated Hüyük-2 as a distinct, highly asymmetric group. Substantial cytogenetic diversity exists among Turkish Lake Region alfalfa populations, including rare features such as B-chromosomes and heteromorphic pairs. These findings highlight the value of local germplasm as genetic resources for conservation and breeding. The Hüyük-2 population, with its highly asymmetric karyotype, is particularly noteworthy for further evolutionary and applied studies.

## Introduction

*Medicago sativa* Linnaeus, 1753 (alfalfa), widely recognized as the “Queen of Forage Crops,” is one of the most important perennial legume species cultivated globally and plays a pivotal role in sustainable animal production systems (Radović et al. 2009). Its significance stems from a combination of superior traits, including high biomass yield, excellent forage quality, broad adaptation, perennial growth habit, and the capacity for biological nitrogen fixation through symbiosis with the nitrogen-fixing bacterium *Ensifer meliloti* (Dangeard 1926; Zhang and Wang 2025). These attributes collectively establish alfalfa as an indispensable source of high-quality roughage for both cattle and small ruminant livestock (Radović et al. 2009; Zhang and Wang 2025). Furthermore, its deep taproot system contributes positively to soil structure, carbon and nitrogen cycling, and enhances resilience to various climatic stresses (Radović et al. 2009).

From a genetic and breeding perspective, alfalfa possesses a complex genomic architecture. Cultivated alfalfa is an autotetraploid species (2n = 4x = 32) characterized by high heterozygosity and an allogamous (cross-pollinated) mating system (Brummer 1999; Yu et al. 2017). This genetic complexity generates substantial variation within and among populations, offering significant opportunities for breeding programs (Eren 2016; Arcia-Ruiz et al. 2025). However, it also presents challenges for conventional methodologies due to polysomic inheritance and inbreeding depression (Brummer 1999). Contemporary breeding efforts are increasingly shaped by techniques such as genomic selection and Genome-Wide Association Studies (GWAS), which have identified molecular markers associated with high biomass yield under drought stress (Medina et al. 2025). The success of such advanced programs depends directly on the identification, characterization, and utilization of diverse genetic resources.

While molecular marker systems are widely used to analyze genetic variation, classical cytogenetic approaches remain fundamental and complementary for determining genome stability and ploidy level. Karyotype analysis provides direct insights into genomic structure by evaluating parameters such as chromosome number, morphology, centromere position, and karyotype symmetry (Peruzzi and Eroğlu 2013). In *Medicago* spp., such analyses are particularly challenging because chromosomes are very small (typically 2–4 µm) (Bauchan and Campbell 1994; Albayrak et al. 2015). Despite this difficulty, detailed karyotyping is essential for identifying cytological features such as structural variations (e.g., heteromorphic pairs, B-chromosomes) and assessing the overall stability of polyploid genomes (Camacho et al. 2000; Jones 2012). Quantitative asymmetry indices, such as the intrachromosomal (A1) and interchromosomal (A2) asymmetry indices, allow objective comparison of karyotype structure and have been widely used to infer evolutionary relationships and cytogenetic differentiation among populations (Zarco 1986; Peruzzi and Eroğlu 2013).

Recent methodological developments have emphasized a standardized set of non-redundant karyomorphological parameters. Peruzzi and Altınordu (2014) proposed mean centromeric asymmetry (MCA), coefficient of variation of chromosome length (CVCL), and coefficient of variation of centromeric index (CVCI) as complementary measures of intrachromosomal asymmetry, interchromosomal chromosome-length variation, and centromere-position heterogeneity, respectively. More recently, Peruzzi et al. (2024) demonstrated that observer-related measurement bias can affect karyomorphological parameters, particularly CVCI and CVCL, emphasizing the importance of a consistent measurement protocol and, where possible, a single observer throughout an investigation.

Populations collected from natural vegetation that have adapted to specific ecological niches over long periods constitute invaluable genetic resources for regional breeding initiatives. These local landraces have evolved under natural selection pressures and may harbor genes associated with tolerance to drought, cold, edaphic factors, and various biotic stresses (Annicchiarico et al. 2015). Identifying genotypes adapted to regions with characteristic semi-arid and transitional climatic features, such as Central Anatolia and the Lake Region of Türkiye, is of paramount importance for selecting parental lines in hybridization, polycross, and synthetic variety development programs.

This study encompasses the collection of alfalfa populations adapted to Central Anatolian conditions from natural vegetation sites in the Afyonkarahisar, Burdur, Isparta and Konya provinces, located within the Lake Region of Türkiye, and the execution of detailed karyotype characterization for these populations. The specific aims were to: (1) determine the somatic chromosome number and detailed karyotype morphology of eight local alfalfa populations, (2) calculate karyotype asymmetry indices (A1, A2) and other descriptive parameters (TF%, DRL, DI), while additionally assessing MCA, CVCL, and CVCI to quantitatively characterize cytogenetic diversity, (3) identify any cytogenetic peculiarities such as B-chromosomes or heteromorphic pairs, and (4) evaluate the cytogenetic similarities and differences among populations to identify those possessing superior genetic attributes for use as genetic resources in future breeding efforts, including hybridization, polycross nurseries, and the development of synthetic cultivars.

## Material and methods

### Taxon sampling

Plant material was collected from eight localities across four provinces of the Lake Region of Türkiye during the growing season (Table 1). Two populations per province were sampled: Yeşilova-3 and Bucak-2 from Burdur; Hüyük-2 and Akşehir-2 from Konya; Uluborlu-1 and Yalvaç-3 from Isparta; and Şuhut-2 and Bolvadin-3 from Afyonkarahisar. Sampling sites were selected to represent the altitudinal and geographical range of the region, spanning elevations from 789 m (Bucak-2) to 1188 m (Uluborlu-1) above sea level. The sampling sites represent diverse ecological conditions within the region. Geographic coordinates were recorded using a GPS device in decimal-degree WGS84 format. Root tips were collected from germinated seeds and from young seedlings that were grown in pots under controlled greenhouse conditions, immediately fixed, and stored for cytological processing.

**Table 1.** Collection localities and geographical coordinates of *Medicago sativa* populations.

**Population Code**	**Locality (Province)**	**Coordinates**	**Altitude (a.s.l.)**
Yeşilova-3	Yeşilova (Burdur)	37.9600°N, 29.7540°E	1152
Bucak-2	Bucak (Burdur)	37.4590°N, 30.5950°E	789
Hüyük-2	Hüyük (Konya)	37.9530°N, 31.5990°E	1130
Akşehir-2	Akşehir (Konya)	38.3570°N, 31.4160°E	1068
Uluborlu-1	Uluborlu (Isparta)	38.0780°N, 30.4500°E	1188
Yalvaç-3	Yalvaç (Isparta)	38.2950°N, 31.1770°E	1182
Şuhut-2	Şuhut (Afyon)	38.5310°N, 30.5450°E	1179
Bolvadin-3	Bolvadin (Afyon)	38.7110°N, 31.0480°E	982

### Plant material preparation for somatic chromosome observation

Cuttings were taken from young shoots of the alfalfa populations and planted in rooting beds containing a perlite/peat (2:1) mixture under mist propagation in a shaded plastic greenhouse to induce rooting. Three rooted cuttings from each location were transplanted into pots and allowed to grow. For cytological analysis, healthy, fresh white root tips (1.5–2.0 cm in length) were excised from the rooted plants. The collected root tips were pretreated in a 1% α-monobromonaphthalene solution at +4 °C for 3 hours. To enhance the penetration of the pretreatment solution into the cells, dimethyl sulfoxide (DMSO) was added, following the method of Mujeeb-Kazi et al. (1987). Following pretreatment, the root tips were fixed in Lewitsky’s fixative (a mixture of chromic acid and formaldehyde) at +4 °C for 30 hours (Agayev et al. 2010). After fixation, the samples were hydrolyzed in 1N sodium hydroxide (NaOH) at 60 °C for 10 minutes (Elçi 1994). Immediately after hydrolysis, the root tips were stained in aceto-iron-hematoxylin at 30–34 °C for 15 hours (Zarifi et al. 2005).

Chromosome preparations were made using the squash technique. The stained root tips were cut to a length of 1.0–1.5 mm and treated with the enzyme Cytase for 2 hours to soften the tissue and macerate the cell walls (Zarifi 2004). The softened root tips were then placed on a microscope slide, a drop of 45% acetic acid (CH_3_COOH) was added, and the tissue was cut into small pieces with a razor blade. The material was covered with a coverslip and gently squashed to spread the cells. The prepared slides were examined under an Olympus BX51 microscope. Metaphase plates from 10–20 cells were evaluated per population, and images of the best 5–10 plates, showing well-spread chromosomes, were captured with a digital camera.

### Karyotype analysis

Chromosome measurements were performed on the captured digital images using MicroMeasure 3.3 software (Reeves 2001). The following parameters were measured for each chromosome: total chromosome length, long arm length, and short arm length. These measurements were used to calculate the arm ratio and the centromeric index. The classification of chromosomes based on centromere position was carried out according to Levan et al. (1964). Chromosomes were classified according to centromere position based on the centromeric index (CI), following the criteria of Levan et al. (1964). The chromosome categories used in the present study were metacentric (m) and submetacentric (sm); no separate near-submetacentric (~sm) category was used. Karyotype symmetry was assessed using the categories defined by Stebbins (1971), and the asymmetry indices (A1 and A2) were calculated according to the method of Blondon et al. (1994).

In accordance with the recommendations of Peruzzi and Altınordu (2014), mean centromeric asymmetry (MCA), coefficient of variation of chromosome length (CVCL), and coefficient of variation of centromeric index (CVCI) were additionally calculated. MCA was calculated as the mean of [(L−S)/(L+S)] × 100, CVCL as [SD(L+S)/mean(L+S)] × 100, and CVCI as [SD{S/(L+S)}/mean{S/(L+S)}] × 100. These parameters quantify, respectively, intrachromosomal asymmetry, interchromosomal variation in chromosome length, and heterogeneity in centromere position. Because the original unrounded MicroMeasure dataset was not available for the revision, MCA, CVCL, and CVCI were reconstructed from the chromosome-pair mean long- and short-arm measurements reported in Suppl. material 1. Therefore, these values should be interpreted as reconstructed estimates based on the reported karyomorphometric data rather than as direct outputs from the original raw MicroMeasure dataset. All chromosome observations and measurements were performed by the same operator to minimize inter-observer variation, following the recommendation of Peruzzi et al. (2024).

## Results

### General karyomorphological features

The chromosomes across all populations were observed to range in size from small to medium. All eight alfalfa populations consistently exhibited a somatic chromosome number of 2n = 4x = 32, thereby confirming their autotetraploid status. The haploid complement length (HCL) demonstrated significant variation among populations, ranging from 20.47 μm in Yeşilova-3 to 32.62 μm in Uluborlu-1. The mean chromosome length varied from 2.56 ± 0.04 μm (Yeşilova-3) to 4.08 ± 0.07 μm (Uluborlu-1). The centromere index (CI) values ranged from 40.14 to 42.63 which is consistent with a predominantly metacentric karyotype composition. All populations consistently exhibited four satellite (SAT) chromosomes, identified by distinct secondary constrictions, typically located on the short arms (chromosome 8 in most cases, with chromosome-specific variation noted in Hüyük-2 and Uluborlu-1). The lengths of the satellites varied among the populations (Table 2). Most chromosomes were metacentric, with a smaller number of submetacentric chromosomes observed. The karyotype formulas, based on the morphology of the 32 somatic chromosomes, are summarized in Table 2. Stebbins’ asymmetry categories were predominantly 2A and 2B, indicating generally symmetric karyotypes with moderate size differences between the largest and smallest chromosomes. Complete karyotype data for each population are summarized in Table 2 and described individually in the following subsections. Detailed chromosome-level measurements are provided in Suppl. material 1.

**Table 2.** Summary of karyotype characteristics of eight *Medicago sativa* populations from the Lake Region of Türkiye. TL: total chromosome length (μm); HCL: haploid complement length (μm); CI: centromere index; SAT: satellite length (μm); CV (%): coefficient of variation of chromosome length; A1: intrachromosomal asymmetry index; A2: interchromosomal asymmetry index; DRL: difference in relative chromosome length; TF (%): total form percentage; DI: dispersion index; SC: Stebbins’ karyotype asymmetry category; MCA (%): mean centromeric asymmetry; CVCL (%): coefficient of variation of chromosome length; CVCI (%): coefficient of variation of centromeric index.

**Population**		**Karyotype formula**	**Mean TL**	**Range**	**HCL**	**CI**	**SAT**	**CV**	**A1**
Burdur	Yeşilova-3	24m+4sm+4sm^sat^	2.56 ± 0.04	1.69–3.69	20.47 ± 0.382	41.45 ± 0.69	0.82	9.60	0.251
	Bucak-2	28m+4sm^sat^+2B	3.14 ± 0.06	2.12–4.57	25.11 ± 0.550	41.83 ± 0.68	1.03	12.60	0.231
Afyonkarahisar	Bolvadin-3	28m+4sm^sat^	2.99 ± 0.05	2.18–4.24	24.00 ± 0.405	42.63 ± 0.90	1.19	13.40	0.208
	Şuhut-2	24m+4sm+4sm^sat^	3.22 ± 0.05	2.54–4.79	25.72 ± 0.439	41.05 ± 0.97	1.36	11.60	0.251
Isparta	Uluborlu-1	20m+8sm+4sm^sat^	4.08 ± 0.07	3.01–5.27	32.62 ± 0.561	41.38 ± 0.91	1.32	10.90	0.242
	Yalvaç-3	24m+4sm+4sm^sat^	3.28 ± 0.07	1.73–5.20	26.21 ± 0.681	41.30 ± 0.76	1.02	12.70	0.255
Konya	Hüyük-2	24m+4sm+4m^sat^	3.31 ± 0.08	2.02–6.22	26.50 ± 0.794	40.89 ± 0.73	2.09	14.90	0.258
	Akşehir-2	28m+4sm^sat^	2.80 ± 0.05	2.22–4.11	22.38 ± 0.367	42.08 ± 0.86	1.09	13.00	0.222
**Population**		**A2**	**DRL**	**TF**	**DI**	**SC**	**MCA**	**CVCL**	**CVCI**
Burdur	Yeşilova-3	0.096	3.15	41.01	4.318	2B	14.80	9.81	9.72
	Bucak-2	0.126	4.63	41.43	3.320	2B	13.46	12.37	7.77
Afyonkarahisar	Bolvadin-3	0.134	5.13	41.58	3.181	2B	11.86	12.69	8.12
	Şuhut-2	0.116	4.83	40.32	3.539	2B	14.95	9.45	10.15
Isparta	Uluborlu-1	0.109	3.75	40.81	3.796	2B	14.58	11.39	11.01
	Yalvaç-3	0.127	5.23	40.56	3.252	2B	15.36	9.95	11.63
Konya	Hüyük-2	0.149	5.48	40.14	2.744	2C	15.31	11.95	9.02
	Akşehir-2	0.130	4.83	41.41	3.237	2A	12.83	8.81	7.87

### Population-specific karyotype descriptions

#### Yeşilova-3 Population

**Figure 1.**
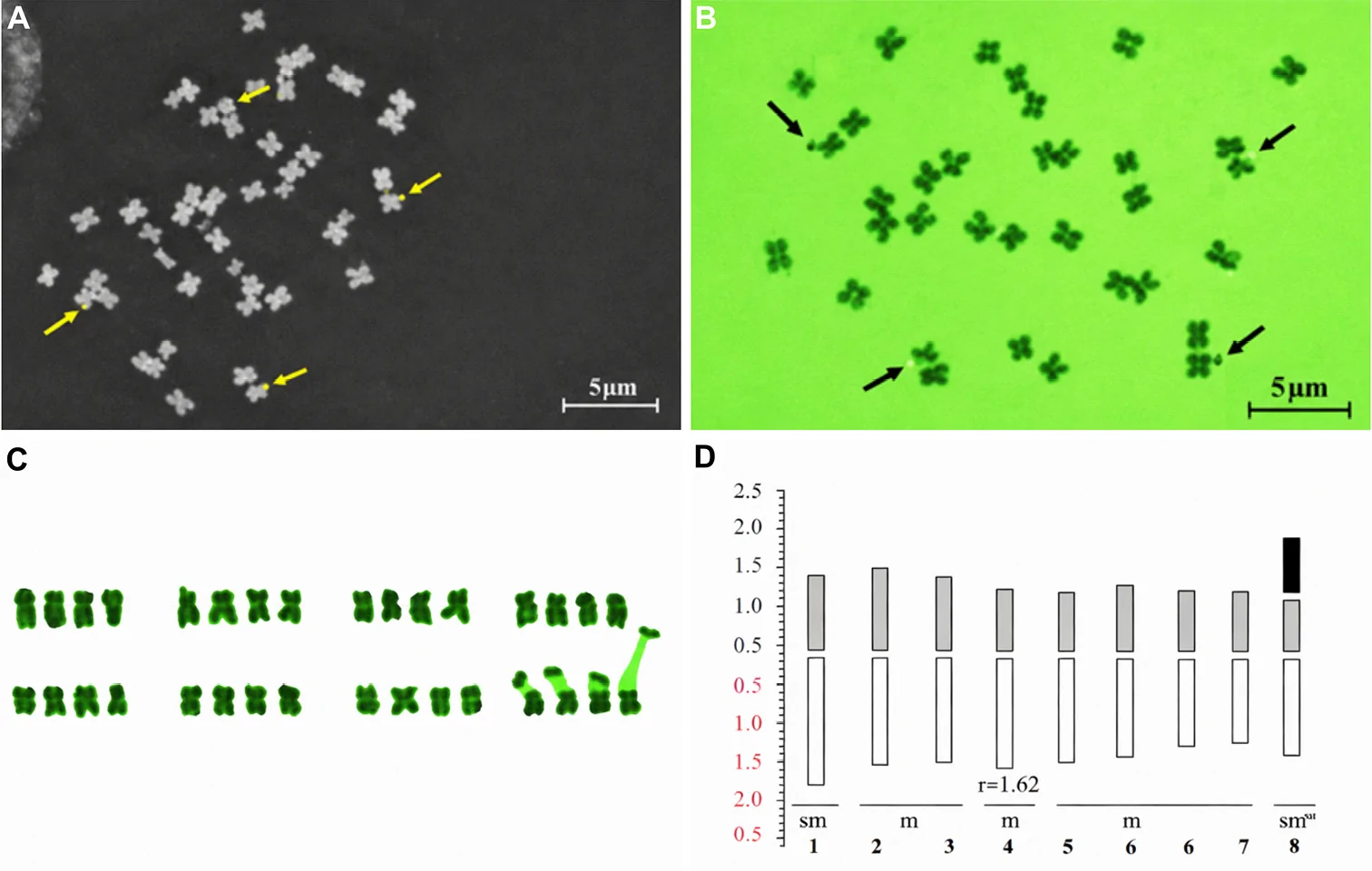
Representative metaphase plates (**A, B**). **C** karyogram (the four satellite-bearing chromosomes are indicated by arrows in all metaphases **D** haploid idiogram of the Yeşilova-3 population.

The karyotype consisted of 24 metacentric, 4 submetacentric, and 4 submetacentric chromosomes bearing satellite (Fig. 1). Chromosome size ranged from 2.21 ± 0.08 μm (smallest) to 2.85 ± 0.12 μm (largest). HCL = 20.47 μm (Table 2, Suppl. material 1). Chromosomes 1 and 8 were submetacentric; the remaining chromosomes were metacentric. Four distinct satellites were clearly visible. The asymmetry indices (A1 = 0.251, A2 = 0.096) and high TF% (41.01) and DI (4.318) indicate a relatively symmetric karyotype with a broad dispersion of chromosome sizes (Fig. 1, Table 2). The reconstructed MCA (14.80%), CVCL (9.81%), and CVCI (9.72%) provide complementary estimates of intrachromosomal asymmetry, chromosome-length variation, and centromere-position heterogeneity, respectively. The karyotype formula was 2n = 4x = 32 = 24m + 4sm + 4sm + 4sm^sat^.

#### Bucak-2 Population

**Figure 2.**
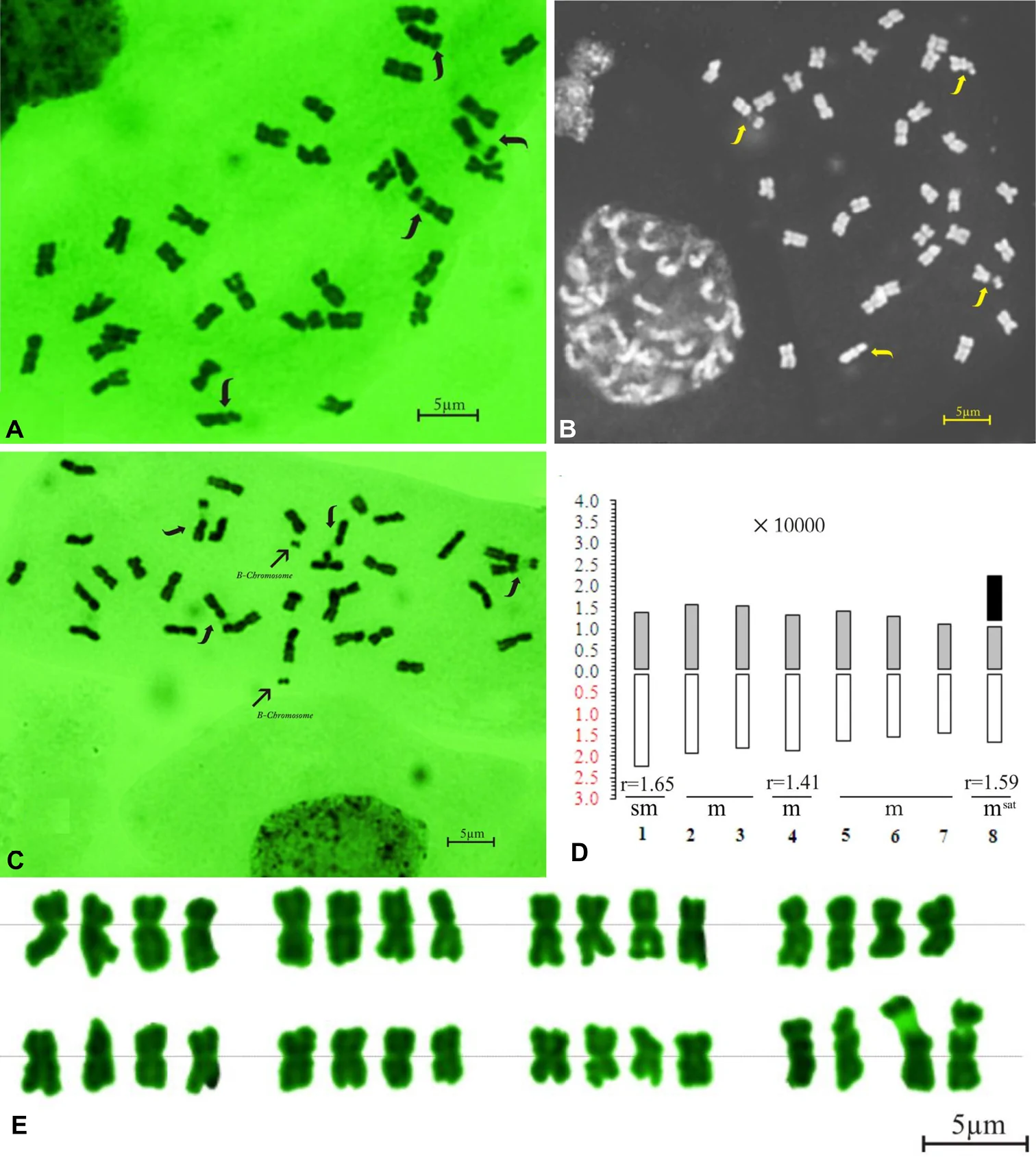
Appearance of chromosomes of the Bucak-2 clover population at mitotic metaphase. **A, B** the four satellite chromosomes are indicated by arrows in all metaphases **C** presence of B-chromosomes in the mitotic metaphase plate **D** haploid idiogram (m: metacentric, sm: sub-metacentric). **E** karyogram, made from the chromosomes of plate A (2n = 4x = 32).

This population was unique due to the presence of B-chromosomes. In addition to the standard 32 A-chromosomes, small metacentric B-chromosomes were consistently observed in all analyzed metaphase plates (2n = 4x = 32 + 2B) (Fig. 2). B-chromosomes were metacentric and morphologically distinct from the standard complement due to their smaller size and dense staining. Chromosome size ranged from 2.49 ± 0.10 μm to 3.52 ± 0.12 μm. Chromosomes were larger than in Yeşilova-3, with a mean length of 3.14 μm and HCL of 25.11 μm (Table 2, Suppl. material 1). The A2 value (0.126) was relatively high, indicating greater variation in chromosome size (Table 2). The reconstructed MCA, CVCL, and CVCI were 13.46%, 12.37%, and 7.77%, respectively. The karyotype consisted of 28 metacentric and 4 submetacentric chromosomes bearing satellites. The karyotype formula was 2n = 4x = 32 = 28m + 4sm^sat^, the two B-chromosomes were recorded separately as supernumerary chromosomes (Table 2, Suppl. material 1).

#### Hüyük-2 Population

**Figure 3.**
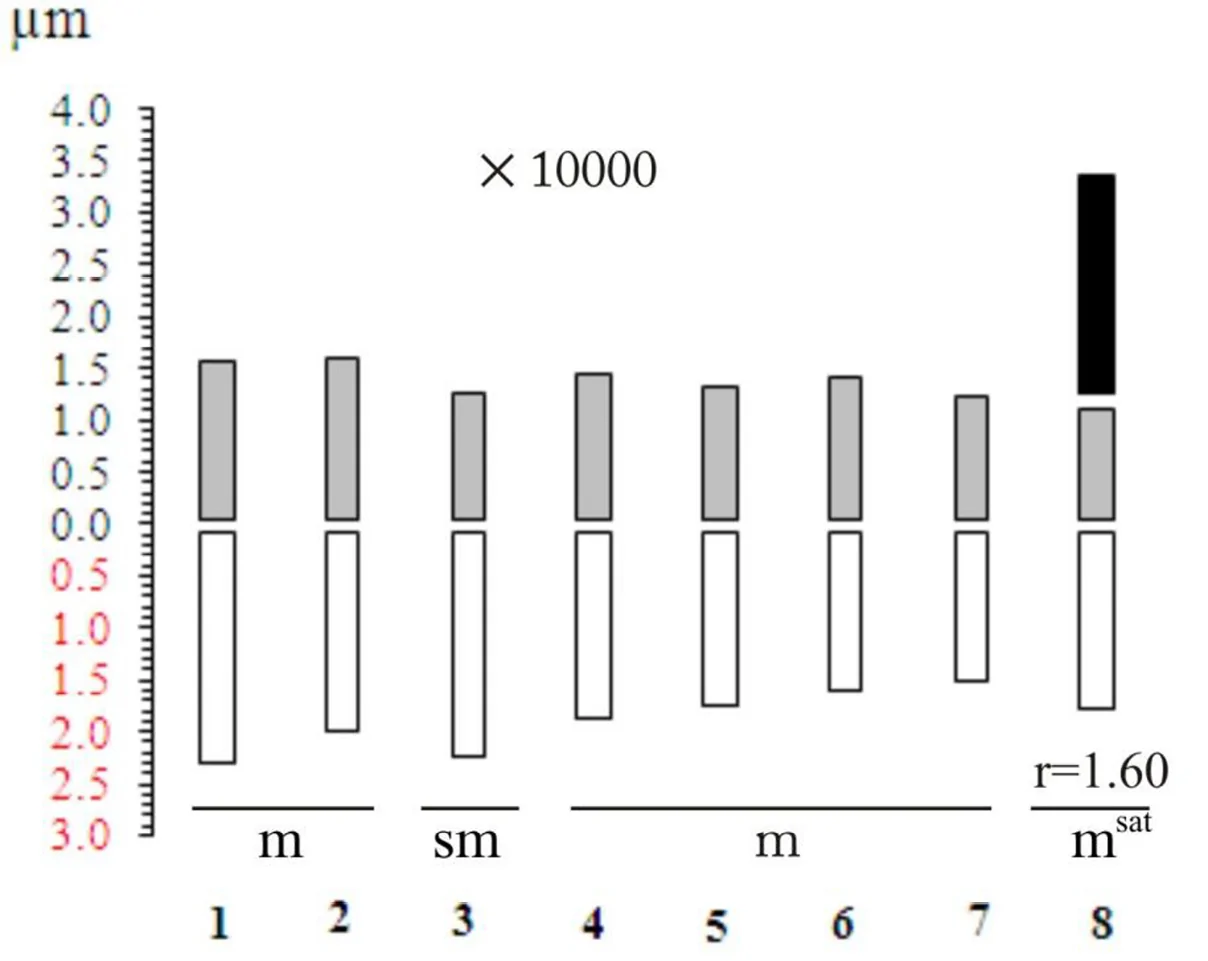
Haploid idiogram of the Hüyük 2 *Medicago sativa* population (2n = 4x = 32). Chromosome types: m = metacentric, sm = submetacentric.

**Figure 4.**
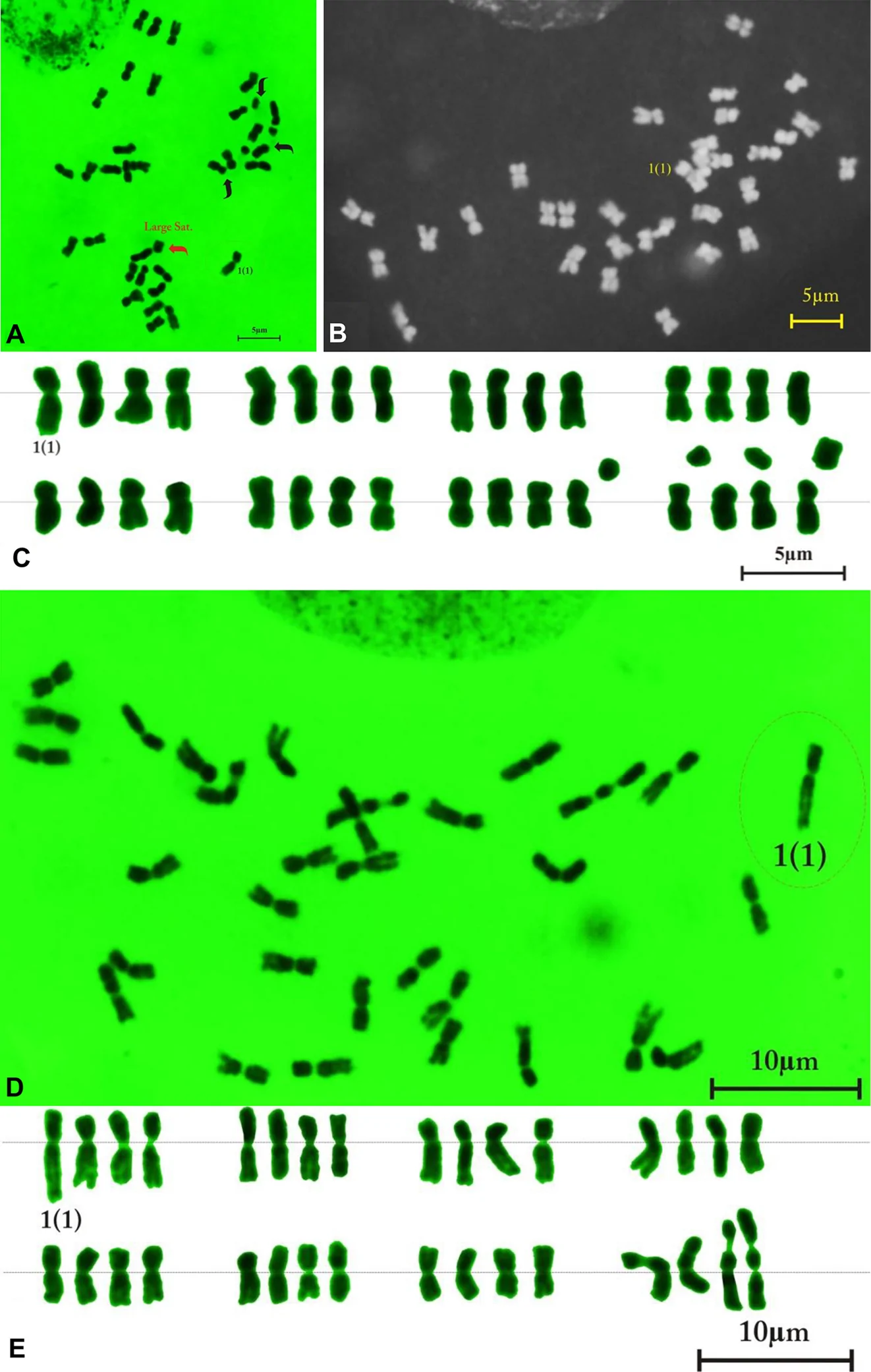
Hüyük-2 *Medicago sativa* population. **A, B** mitotic metaphase plates **C** karyogram constructed from the metaphase plate shown in A (2n = 4x = 32); the four satellite-bearing chromosomes and the heteromorphic chromosome are indicated by arrows **D** mitotic metaphase spread **E** karyogram showing details of the heteromorphic chromosome.

This population displayed the most asymmetric karyotype overall, with the highest A1 (0.258) and A2 (0.149) values, and was the only population classified under Stebbins’ 2C category (Fig. 4, Table 2). Chromosome size ranged from 2.65 ± 0.15 μm to 4.10 ± 0.27 μm, representing the widest size range. HCL = 26.50 μm (Table 2, Suppl. material 1). Chromosome 3 was submetacentric. Two remarkable cytogenetic features distinguished this population: (i) chromosome 8 possessed an unusually large satellite on its short arm, approximately equal in length to the short arm itself (Fig. 3); and (ii) heteromorphic chromosomes were unambiguously identified in the first pair, with the two homologs differing in both total length and arm ratio (Fig. 4). The reconstructed MCA was 15.31%, while CVCL was 11.95% and CVCI was 9.02%. The high A1 and A2 values, together with the relatively high CVCL, support the interpretation of Hüyük-2 as the most structurally distinctive population. The karyotype formula was 24m + 4sm + 4m^sat^ (Table 2, Suppl. material 1).

#### Akşehir-2 Population

**Figure 5.**
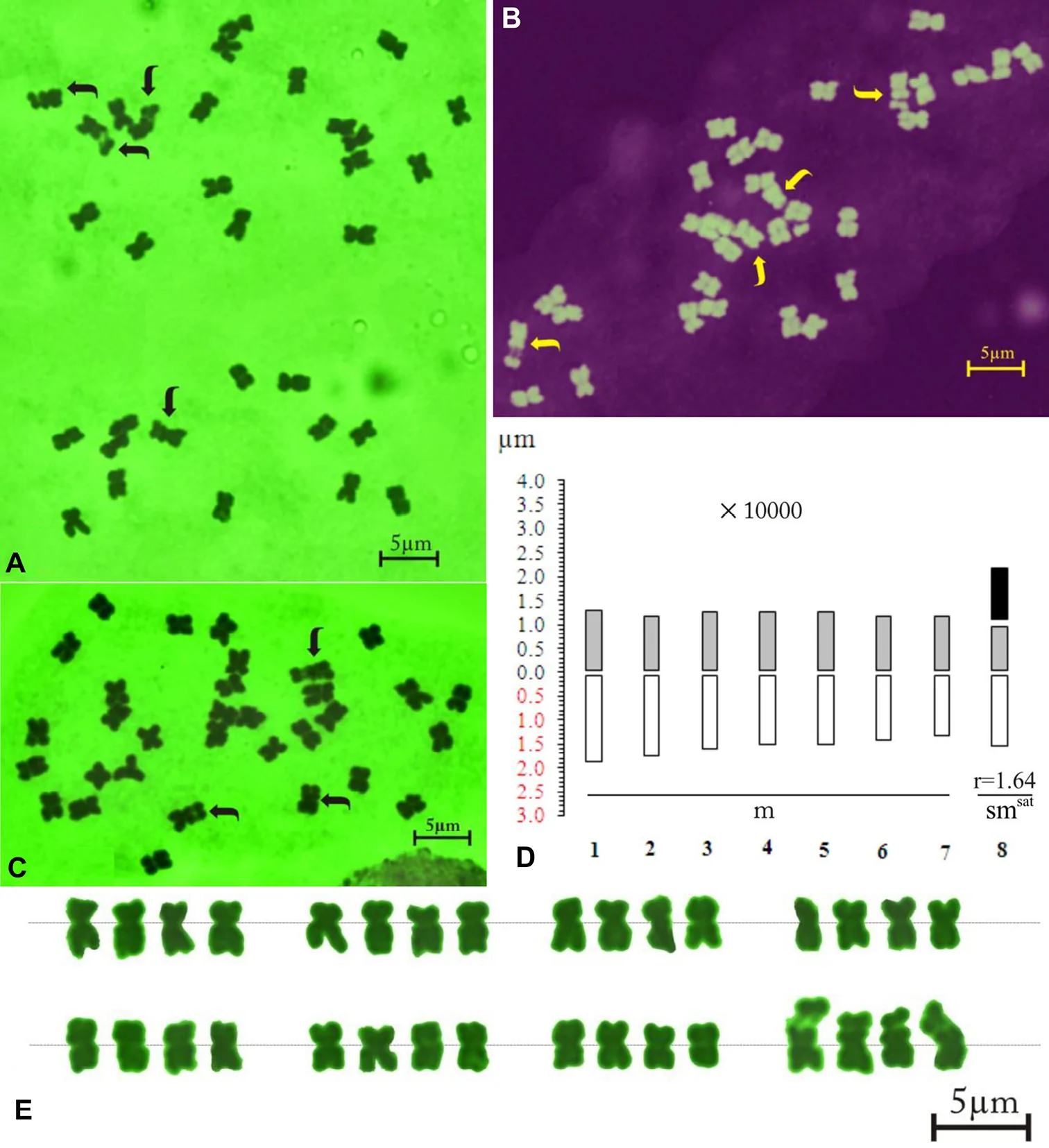
Akşehir-2 *Medicago sativa* population. **A–C** mitotic metaphase plates **D** haploid idiogram (m: metacentric, sm: submetacentric) **E** karyogram constructed from the metaphase plate shown in A (2n = 4x = 32). The four satellite-bearing chromosomes are indicated by arrows in all metaphases.

The karyotype (28m + 4sm^sat^) was similar to Bolvadin-3 and Yalvaç-3, being predominantly metacentric (Fig. 5). The A1 (0.222) and A2 (0.130) values were intermediate, and the SC category was 2A (Table 2), indicating a symmetric karyotype with a small size difference between the largest and smallest chromosomes. Chromosome size ranged from 2.39 ± 0.04 μm to 3.47 ± 0.17 μm (Fig. 5, Suppl. material 1). The reconstructed MCA, CVCL, and CVCI were 12.83%, 8.81%, and 7.87%, respectively, indicating comparatively low intrachromosomal and centromere-position variation among the populations examined (Table 2, Suppl. material 1).

#### Uluborlu-1 Population

**Figure 6.**
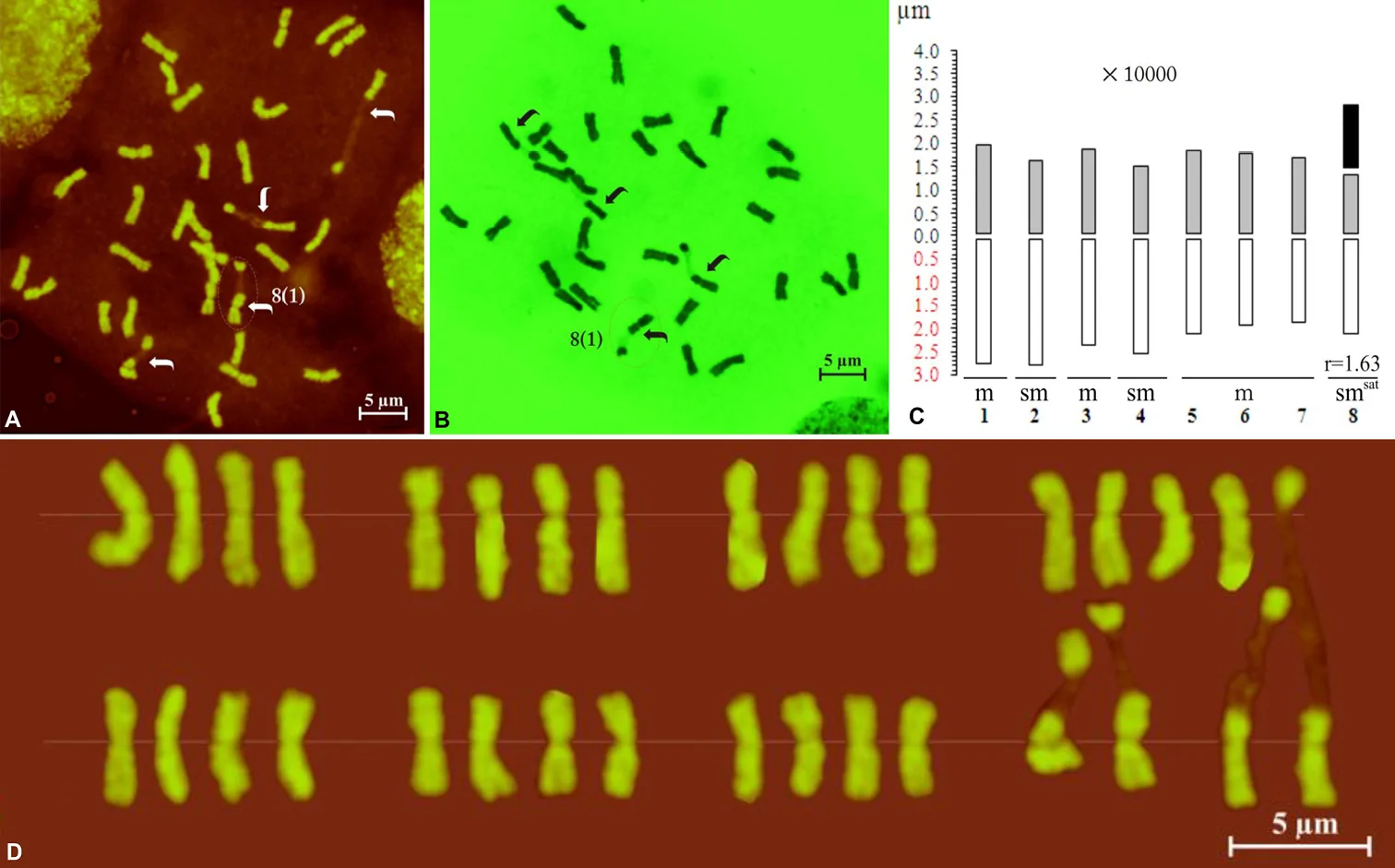
Uluborlu-1 *Medicago sativa* population. **A, B** mitotic metaphase plates **C** haploid idiogram (m: metacentric, sm: submetacentric) **D** karyogram constructed from the metaphase plate shown in A (2n = 4x = 32). The four satellite-bearing chromosomes and the heteromorphic chromosome are indicated by arrows in all metaphases.

This population exhibited the largest chromosomes overall (mean TL = 4.08 μm; TC = 32.62 μm) (Table 2, Suppl. material 1). The karyotype consisted of 20 metacentric, 8 submetacentric, and 4 submetacentric chromosomes bearing satellites. The karyotype formula was 2n = 4x = 32 = 20m + 8sm + 4sm^sat^ (Fig. 6). A heteromorphic chromosome pair (likely chromosome 1) was also observed, where homologous chromosomes differed in size or arm ratio (Fig. 6). The A1 value (0.242) was moderate, but the relatively low A2 (0.109) suggests that interchromosomal size variation was not extreme according to the original A2 measure. The reconstructed MCA, CVCL, and CVCI were 14.58%, 11.39%, and 11.01%, respectively. The relatively high CVCI indicates appreciable heterogeneity in centromere position, consistent with the presence of several submetacentric chromosomes (Table 2).

#### Yalvaç-3 Population

**Figure 7.**
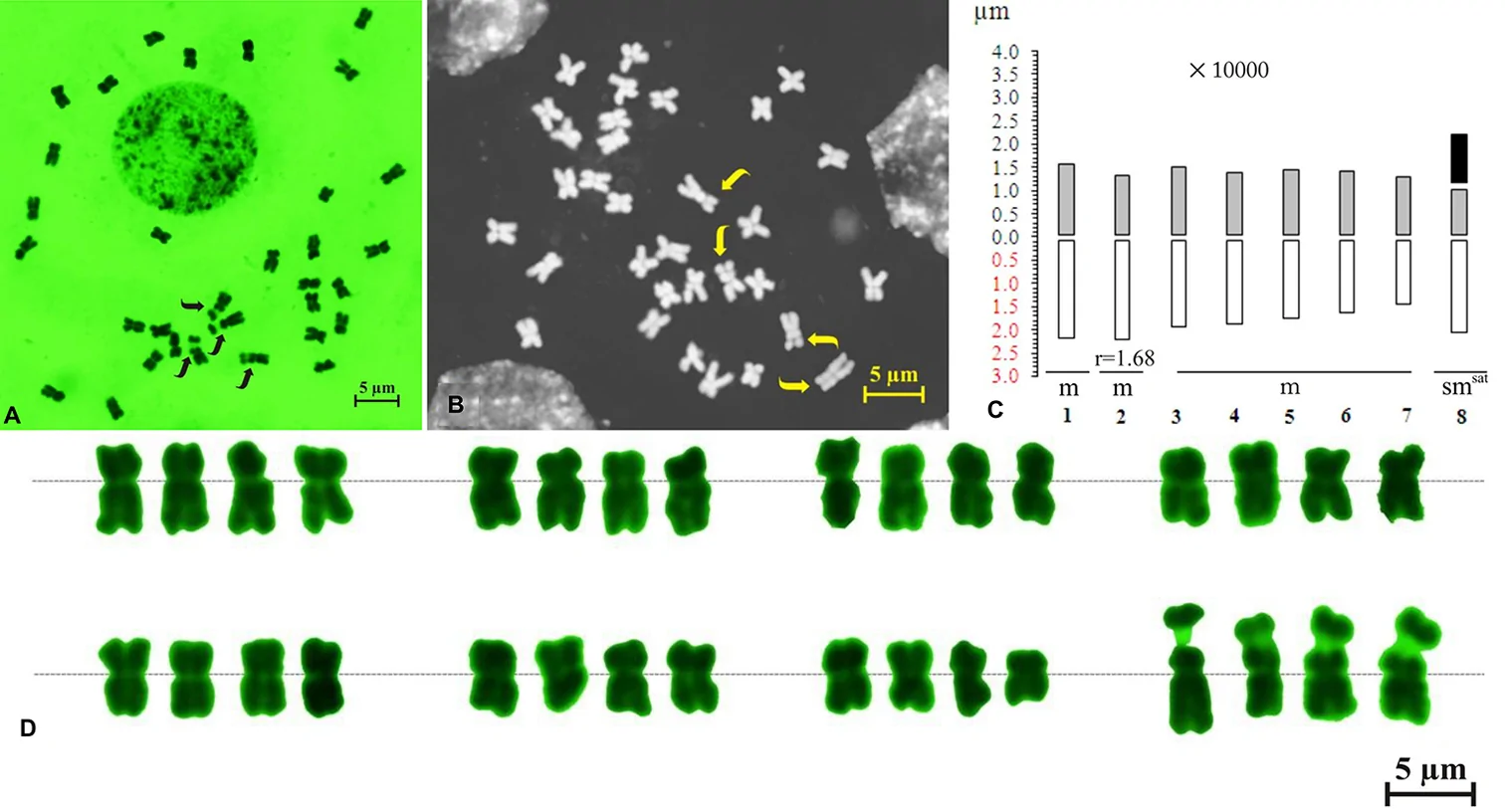
Yalvaç-3 *Medicago sativa* population. **A, B** mitotic metaphase plates **C** haploid idiogram (m: metacentric, sm: submetacentric) **D** karyogram constructed from the metaphase plate shown in A (2n = 4x = 32). The four satellite-bearing chromosomes are indicated by arrows in all metaphases.

This population had a relatively symmetric karyotype (24m + 4sm + 4sm^sat^) with a high proportion of metacentric chromosomes (Fig. 7). Chromosome size ranged from 2.62 ± 0.14 μm to 3.99 ± 0.21 μm. HCL = 26.21 μm (Table 2, Suppl. material 1). Chromosomes 2 and 8 were submetacentric, all others were metacentric. The karyotype formula was 2n = 4x = 32 = 24m + 4sm + 4sm^sat^, SC = 2B, A1 = 0.255, A2 = 0.127. The A2 and DRL values (5.23) were among the highest recorded, indicating substantial interchromosomal size variation (Table 2). The reconstructed MCA (15.36%) was the highest among the eight populations, and CVCI (11.63%) was also the highest, indicating comparatively pronounced intrachromosomal asymmetry and centromere-position heterogeneity despite the predominance of metacentric chromosomes (Table 2).

#### Şuhut-2 Population

**Figure 8.**
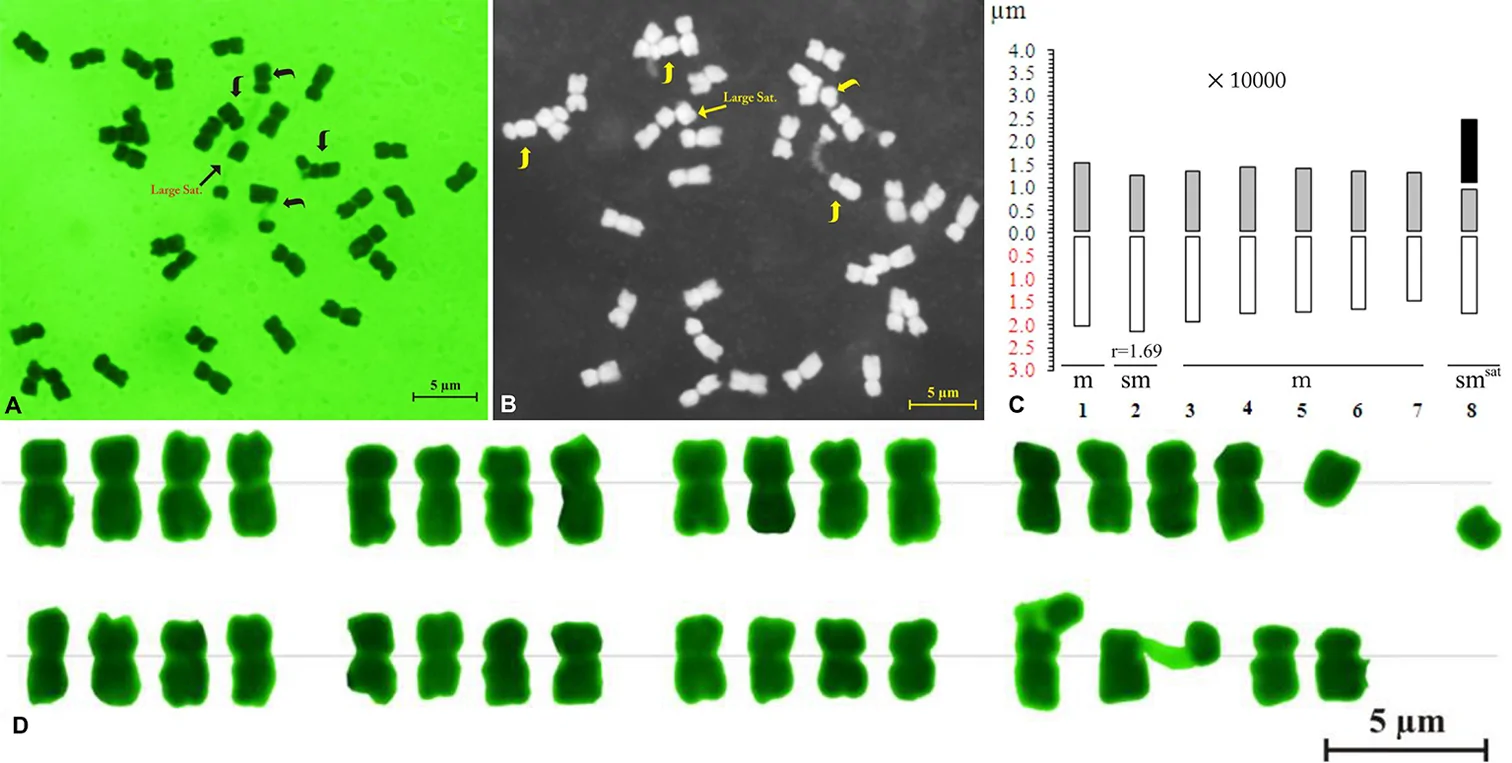
Şuhut-2 *Medicago sativa* population. **A, B** mitotic metaphase plates **C** haploid idiogram (m: metacentric, sm: submetacentric) **D** karyogram constructed from the metaphase plate shown in A (2n = 4x = 32). The four satellite-bearing chromosomes are indicated by arrows in all metaphases.

Chromosome size ranged from 2.72 ± 0.06 μm to 3.96 ± 0.16 μm. HCL = 25.72 μm (Table 2, Suppl. material 1). The karyotype (24m + 4sm + 4sm^sat^) was characterized by a distinct submetacentric pair (chromosome 2) in addition to the satellite-bearing chromosome 8 (Fig. 8). The asymmetry indices (A1 = 0.251, A2 = 0.116) and TF% (40.32) were moderate. The reconstructed MCA, CVCL, and CVCI were 14.95%, 9.45%, and 10.15%, respectively (Table 2).

#### Bolvadin-3 Population

**Figure 9.**
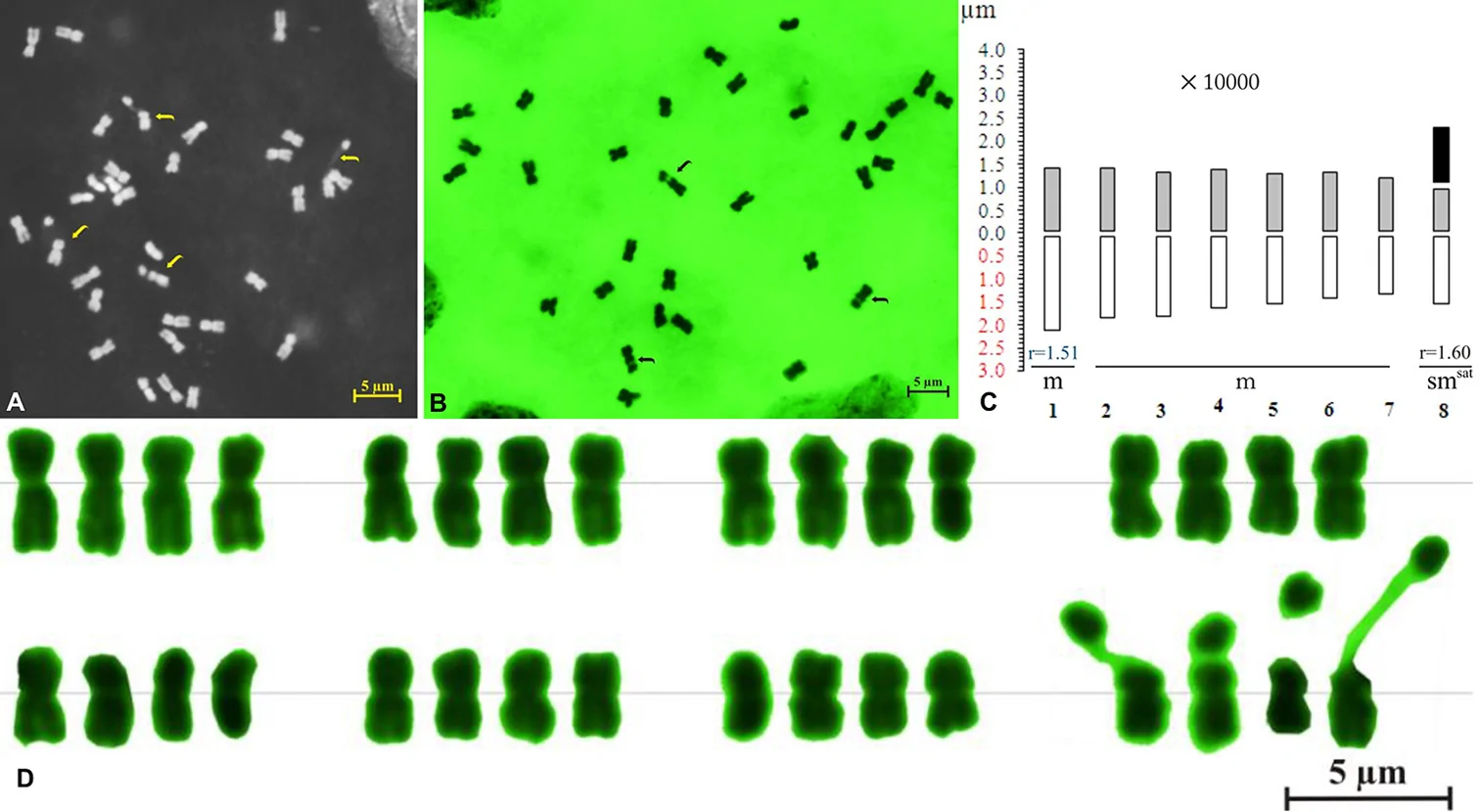
Bolvadin-3 *Medicago sativa* population. **A, B** mitotic metaphase plates **C** haploid idiogram (m: metacentric, sm: submetacentric) **D** karyogram constructed from the metaphase plate shown in A (2n = 4x = 32). The four satellite-bearing chromosomes are indicated by arrows in all metaphases.

The karyotype (28m + 4sm^sat^) was predominantly metacentric, with four submetacentric chromosomes bearing satellites (Fig. 9). The A1 (0.208) and A2 (0.138) values were the lowest and relatively high, respectively, among the populations examined (Table 2). Chromosome size ranged from 2.41 ± 0.05 μm to 3.33 ± 0.09 μm (Fig. 9, Suppl. material 1). The reconstructed MCA, CVCL, and CVCI were 11.86%, 12.69%, and 8.34%, respectively (Table 2, Suppl. material 1).

### Karyotype asymmetry and population relationships

**Figure 10.**
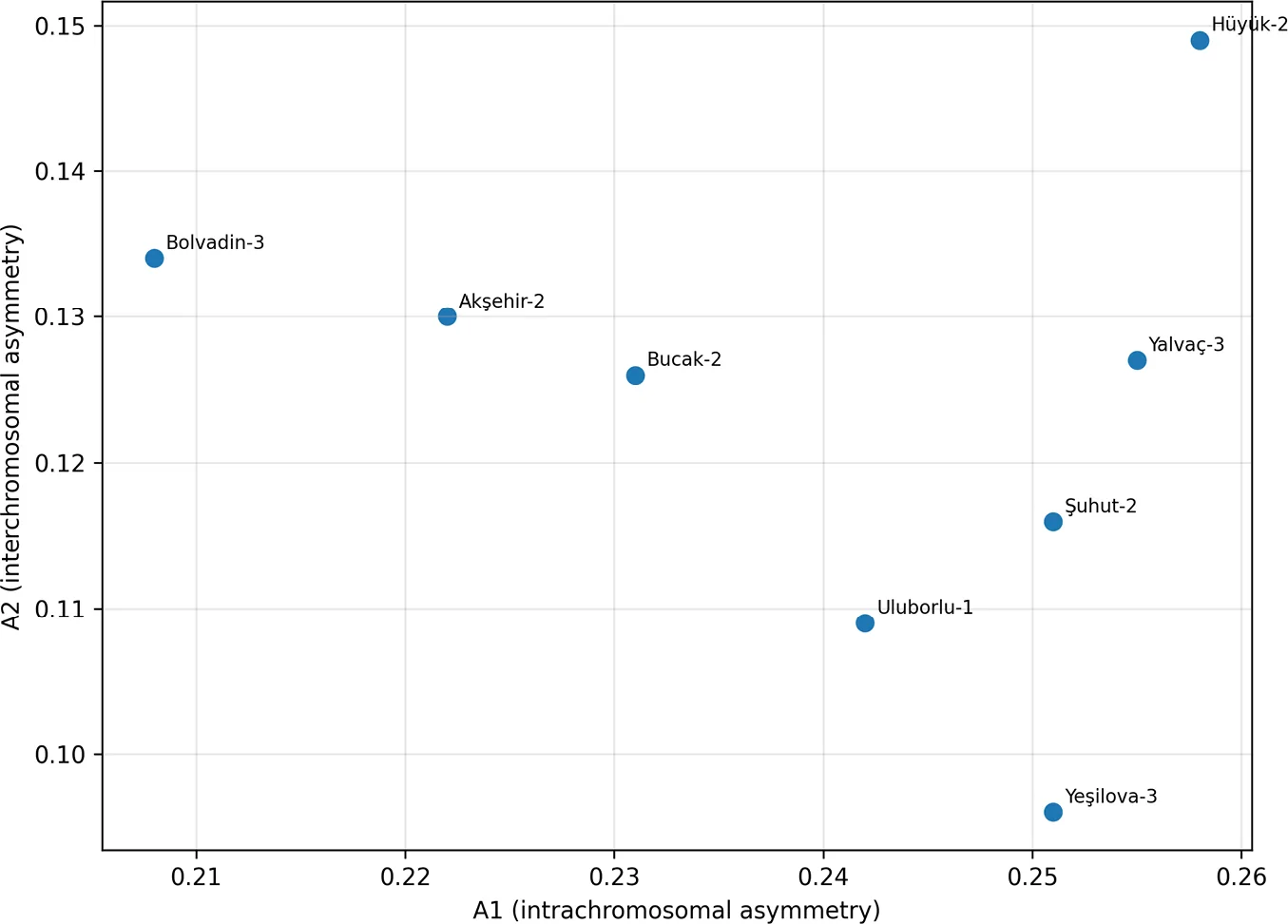
Karyotype asymmetry distribution of *Medicago sativa* populations based on A1 and A2 indices.

The A1 and A2 indices revealed a spectrum of karyotype asymmetry among the populations. The scatter plot (Fig. 10) visually separates populations based on these two dimensions. Hüyük-2 stands out with the highest values for both indices. In contrast, Yeşilova-3 and Bolvadin-3 occupy positions reflecting lower asymmetry, with Bolvadin-3 showing the lowest A1 and Yeşilova-3 the lowest A2.

The newly reconstructed MCA, CVCL, and CVCI values further resolved different components of karyotype variation. MCA ranged from 11.86% in Bolvadin-3 to 15.36% in Yalvaç-3. CVCL ranged from 8.81% in Akşehir-2 to 12.69% in Bolvadin-3, whereas CVCI ranged from 7.77% in Bucak-2 to 11.63% in Yalvaç-3. Hüyük-2 remained distinctive because of its high A1 and A2 values and its Stebbins 2C classification. Because the reconstructed indices were derived from rounded chromosome-pair means, they are interpreted here as complementary descriptive measures rather than replacements for the original A1/A2 analysis.

**Figure 11.**
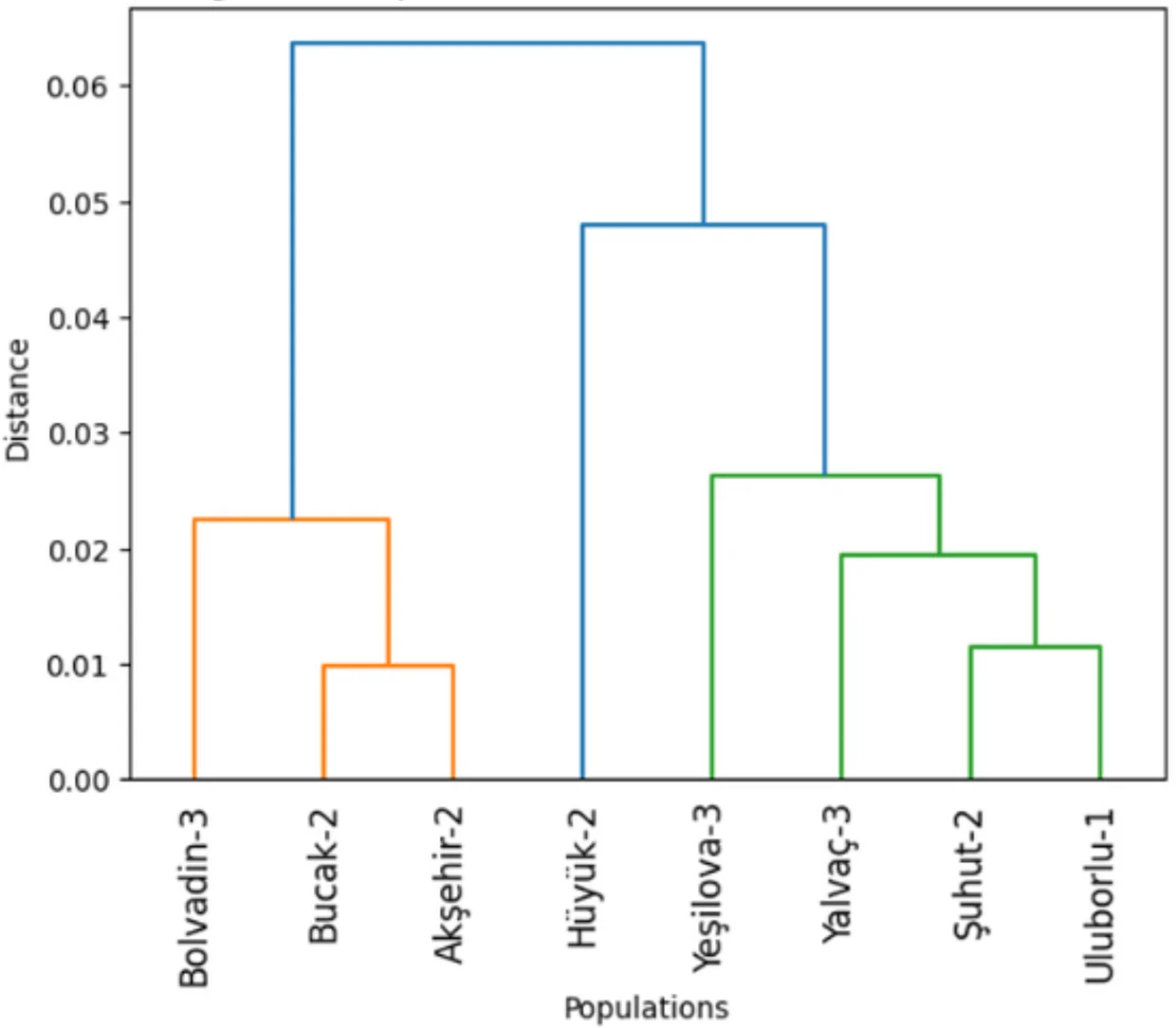
Dendrogram showing cytogenetic similarity among eight populations of *Medicago sativa* based on A1 and A2 data.

The cluster analysis based on A1 and A2 (Fig. 11) further elucidated these relationships. Populations were grouped into distinct clusters. A primary cluster included Yeşilova-3, Şuhut-2, Uluborlu-1, and Yalvaç-3, showing moderate similarity in their asymmetry indices. Another cluster grouped Bucak-2, Bolvadin-3, and Akşehir-2, which generally exhibited more symmetric karyotypes. Notably, Hüyük-2 formed a separate, distinct branch, confirming its unique, highly asymmetric karyotype structure.

The cluster analysis was retained as an A1/A2-based analysis; the newly added MCA, CVCL, and CVCI parameters were not used to reconstruct the dendrogram because the original clustering procedure was based specifically on A1 and A2. This preserves comparability with the submitted analysis while providing the reviewer-requested standardized descriptors in the population-level results.

## Discussion

This study provides a comprehensive karyotype analysis of eight local alfalfa populations from the Lake Region of Türkiye, revealing substantial cytogenetic diversity. Our results confirm the autotetraploid genome constitution (2n = 4x = 32, x = 8) in all populations, consistent with extensive prior literature on cultivated alfalfa (Blondon et al. 1994; Yu et al. 2017). The chromosome counts reported here align closely with those reported for tetraploid alfalfa accessions by Bauchan and Campbell (1994) and Özkan (2021) supporting the consistency of our chromosome preparation and chromosome-counting procedures.

Chromosome sizes across all populations (mean: 2.56–4.08 μm) are consistent with the characteristically small chromosomes of *Medicago* species, which typically range between 2 and 4 μm (Albayrak et al. 2015). The hematoxylin staining protocol employed in the present study proved effective in resolving individual chromosomes in compact metaphase arrays, an approach also endorsed by Agayev et al. (2010) and Zarifi et al. (2005), and was also effective for species with small chromosomes. The substantial among-population variation in HCL (20.47–32.62 μm) indicates marked variation in total chromosome complement length among the sampled populations, although this should not be interpreted as direct evidence of genome-size polymorphism. Direct assessment of nuclear DNA content, for example by flow cytometry, would be required to determine whether the observed differences in chromosome complement length are accompanied by differences in genome size. A limitation of the present study is that the chromosome preparations obtained during the original experimental work were not analyzed separately as independent experimental replicates. Although multiple metaphase plates were evaluated for each population, chromosome condensation and slide preparation may introduce variation in chromosome measurements. Because the original preparations, digital images, and unrounded MicroMeasure datasets were no longer available during the revision, a retrospective replicate-based assessment of measurement reproducibility was not possible. Therefore, the observed differences in chromosome complement length should be interpreted with appropriate caution. Future studies should incorporate independent chromosome preparations and replicate measurements to further validate population-level differences in chromosome length.

The predominance of metacentric chromosomes, with a minority of submetacentric elements, mirrors earlier reports for tetraploid alfalfa (Falistocco 2020; Özkan 2021) and is consistent with the predominantly symmetric karyotype expected in a species with x = 8 and a relatively recent allopolyploidization event. However, because chromosome classification in the present study was based on conventional karyomorphometric features, including relative chromosome length, arm ratio, centromeric index, and centromere position, chromosomes with very similar size and morphology cannot always be unequivocally distinguished. Molecular cytogenetic approaches, such as fluorescence *in situ* hybridization (FISH) with chromosome- or locus-specific markers, would provide additional landmarks for precise chromosome identification and homolog discrimination. Such analyses were beyond the scope of the present study and should be considered in future studies of the Turkish alfalfa populations. Stebbins (1971) proposed that symmetric karyotypes are ancestrally primitive, whereas asymmetric karyotypes arise through successive structural rearrangements. The dominance of SC 2B in our populations, with a single SC 2A (Akşehir-2) and a single SC 2C (Hüyük-2), implies that the Lake Region populations occupy an intermediate position in the karyotypic evolutionary gradient, with Hüyük-2 with Hüyük-2 showing the most asymmetric karyotypic profile among the sampled populations.

The presence of four satellite chromosomes in all populations is a notable and consistent feature. This likely corresponds to the four homologous copies of the NOR-bearing chromosome expected in an autotetraploid complement; however, molecular cytogenetic markers would be required to confirm chromosome identity and NOR localization. Recent molecular cytogenetic studies have highlighted the abundance and variability of satellite DNA in *Medicago* (Falistocco 2020), and our results provide a karyotypic basis for these molecular observations.

A key finding of this study is the discovery of B-chromosomes (2n = 4x = 32 + 2B) in the Bucak-2 population. B-chromosomes are supernumerary elements not essential for normal growth and reproduction, often varying in number and morphology (Jones 2012). Their presence in *Medicago* spp. has been previously documented by Bauchan and Hossain (1999). The consistent observation of two small, metacentric B-chromosomes in all examined cells of Bucak-2 suggests they are stably maintained in this population. While their functional role remains unclear, their presence contributes to the overall cytogenetic diversity and may influence recombination or adaptation (Camacho et al. 2000).

Another remarkable finding is the presence of heteromorphic chromosome pairs in the Hüyük-2 and Uluborlu-1 populations. Heteromorphism, where homologous chromosomes differ in size or morphology, is often the result of structural rearrangements such as duplications, deletions, inversions, or unequal translocations (Sadeghian and Hejazi 2014). In outcrossing species like alfalfa, which harbor high levels of genetic variation (Brummer 1999), such structural polymorphisms can become established within populations. The heteromorphic pair in Hüyük-2 was particularly striking, involving a size difference between homologs of chromosome 1 and an unusually large satellite on chromosome 8. This may indicate karyotypic restructuring in the Hüyük-2 population, consistent with its high A1 and A2 values and its placement in the more asymmetric Stebbins 2C category.

The karyotype asymmetry analysis using A1 and A2 indices (Zarco 1986) revealed significant interpopulation variation. A1 values ranged from 0.208 (Bolvadin-3) to 0.258 (Hüyük-2), and A2 values from 0.096 (Yeşilova-3) to 0.149 (Hüyük-2). Comparable inter-population variation in A1 and A2 has been documented in other polyploid plant genera (Peruzzi and Eroğlu 2013), and the pattern observed here is broadly consistent with the magnitude of variation reported for natural forage grass and legume populations. The DI values further supported the ranking of populations by karyotype symmetry: Yeşilova-3 showed the highest DI (4.318), indicating a wide dispersion of chromosome sizes, whereas Hüyük-2 displayed the lowest DI (2.744), paradoxically reflecting a more compressed but internally asymmetric size distribution. The complementary TF%, CV%, and DRL metrics consistently positioned Hüyük-2 as the most asymmetric and cytogenetically derived population. The newly introduced MCA values ranged from 11.86% in Bolvadin-3 to 15.36% in Yalvaç-3, indicating that the populations with the lowest and highest MCA were not identical to those identified solely by A1. This illustrates the complementary nature of the parameters: MCA reflects intrachromosomal asymmetry, CVCL reflects interchromosomal chromosome-length variation, and CVCI reflects centromere-position heterogeneity (Peruzzi and Altınordu 2014).

The reconstructed CVCL values ranged from 8.81% in Akşehir-2 to 12.69% in Bolvadin-3, while CVCI ranged from 7.77% in Bucak-2 to 11.63% in Yalvaç-3. The relatively high CVCI of Yalvaç-3 indicates greater heterogeneity in centromere position, despite its predominantly metacentric karyotype. Hüyük-2 remained distinctive because of its high original A1 and A2 values, heteromorphic chromosome pair, enlarged satellite, and 2C classification. Since the newly reconstructed MCA, CVCL, and CVCI values were derived from rounded chromosome-pair means rather than the original raw measurements, these indices are interpreted conservatively. Peruzzi et al. (2024) showed that CVCL and CVCI can be particularly sensitive to observer-related measurement bias, reinforcing the importance of consistent measurement procedures and supporting the use of a single operator in the present study.

Cluster analysis based on A1 and A2 indices grouped populations into three distinct clades. The divergence of Hüyük-2 as a singleton is the most striking pattern and is attributable to its unique combination of heteromorphic chromosomes, an enlarged satellite on chromosome 8, and elevated A1/A2 asymmetry. The grouping of Bolvadin-3 and Akşehir-2 in a symmetric clade, and the clustering of the remaining five populations, broadly reflects geographical proximity but is not entirely concordant with it, Uluborlu-1 (Isparta) and Yalvaç-3 (Isparta) do not co-cluster, suggesting that altitude and local ecological conditions may exert selection pressure on karyotypic characters independently of geographic distance. This observation warrants further investigation using molecular markers to disentangle drift, gene flow, and adaptation.

From an applied perspective, the cytogenetic diversity revealed in this study has direct implications for alfalfa improvement programs in Türkiye. Populations exhibiting distinct karyotype formulas, elevated chromosome sizes (Uluborlu-1), or unusual chromosomal features (Bucak-2, Hüyük-2) may harbor unique allelic configurations that are inaccessible from commercially standardized cultivars. The integration of karyotypic data with agromorphological and molecular marker characterization will be essential for prioritizing accessions for conservation and crossing schemes (Monirifar and Moharrmanejad 2021). Moreover, the persistence of B-chromosomes in Bucak-2 raises questions regarding the ecological stability of this germplasm and its potential use in genetic studies of B-chromosome biology in legumes.

In conclusion, the Lake Region of Türkiye harbors substantial cytogenetic diversity in local alfalfa germplasm. The substantial cytogenetic diversity observed in this study has important implications for alfalfa breeding and conservation. Local populations often harbor unique adaptations and genetic resources. The presence of B-chromosomes and heteromorphic pairs, as seen in Bucak-2 and Hüyük-2, may indicate specific local adaptations or unique evolutionary histories. This variation represents a valuable genetic reservoir. In breeding programs, knowledge of karyotype structure and compatibility can guide the selection of parents for crossing, as similar chromosome complements are more likely to result in successful recombination (Monirifar and Moharrmanejad 2021). Therefore, the detailed cytogenetic characterization of these local populations provides crucial baseline data for their conservation and potential utilization in future alfalfa improvement efforts.

## Conclusion

This study presents the first comprehensive comparative cytogenetic characterization of alfalfa populations from the four provinces that comprise Türkiye’s Lake Region. All eight populations were confirmed as autotetraploid (2n = 4x = 32). Despite a generally symmetric karyotype structure dominated by metacentric chromosomes, significant cytogenetic diversity was uncovered through the analysis of karyotype formulas, asymmetry indices (A1, A2), and other descriptive parameters. The populations varied in mean chromosome length, total haploid length, and the number of submetacentric chromosomes. Notably, B-chromosomes were identified in the Bucak-2 population, and heteromorphic chromosome pairs were observed in the Hüyük-2 and Uluborlu-1 populations, indicating ongoing karyotype evolution. Hüyük-2 population stood out as the most structurally distinctive population, whereas Yalvaç-3 showed the highest reconstructed MCA and CVCI values. The complementary use of MCA, CVCL, and CVCI provided separate information on intrachromosomal asymmetry, chromosome-length variation, and centromere-position heterogeneity. Because the original raw MicroMeasure dataset was unavailable, these three indices were reconstructed from the reported chromosome-pair means and should therefore be interpreted as complementary reconstructed descriptors. Nevertheless, their inclusion strengthens the standardized karyomorphometric characterization of the Turkish alfalfa populations and provides a useful foundation for future conservation and breeding studies.

## Additional information

### Artificial Intelligence (AI) use

The author accepts full responsibility for the content of the manuscript, including the disclosure of any use of AI.

No AI tools were used in the preparation of this manuscript.

### Author ORCIDs

D. Güloğlu https://orcid.org/0000-0002-1839-8710

### Data availability

All of the data that support the findings of this study are available in the main text or Supplementary Information.

## Supplementary materials

Supplementary material 1 Detailed chromosome measurements of the eight *Medicago sativa* populations from the Lake Region of Türkiye Data type docx https://binary.pensoft.net/file/1773325This dataset is made available under the Open Database License (http://opendatacommons.org/licenses/odbl/1.0/). The Open Database License (ODbL) is a license agreement intended to allow users to freely share, modify, and use this Dataset while maintaining this same freedom for others, provided that the original source and author(s) are credited. Derya Güloğlu
