# Supplementary material for: Karyotype characterization and cytogenetic diversity of Medicago sativa L. (Leguminosae) populations from the Lake Region of Türkiye
Source: Comp Cytogenet. 2026 Sep 16;20:207–28. doi: 10.3897/compcytogen.20.205237 (PMC13601921; doi:10.3897/compcytogen.20.205237)
Supplement: Supplementary file 1 [file comparative_cytogenetics-20-207_article-205237__-s001.docx]

**Supplementary Table S1. Detailed chromosome measurements of the eight *Medicago sativa* populations from the Lake Region of Turkey.** L = long arm length (µm), S = short arm length (µm), CI = centromeric index, AR = arm ratio, Sat = satellite length (µm), L% and S% = percentage of each arm relative to the total haploid complement length, and RL% = relative chromosome length.

| **Population** | **Pair No.** | **Total chromosome length µm** | **Long arm (L) µm** | **Short arm (S) µm** | **AR** | **CI** | **Sat** | **Type** | **L%** | **S%** | **RL%** |
| --- | --- | --- | --- | --- | --- | --- | --- | --- | --- | --- | --- |
| Yeşilova-3 | 1 | 2.85 | 1.8 | 1.05 | 1.71 | 37 | – | sm | 8.77 | 5.14 | 13.91 |
|  | 2 | 2.71 | 1.49 | 1.22 | 1.23 | 45 | – | m | 7.28 | 5.95 | 13.23 |
|  | 3 | 2.6 | 1.45 | 1.15 | 1.27 | 44 | – | m | 7.1 | 5.61 | 12.71 |
|  | 4 | 2.52 | 1.56 | 0.97 | 1.62 | 38 | – | m | 7.61 | 4.71 | 12.32 |
|  | 5 | 2.41 | 1.31 | 1.1 | 1.23 | 45 | – | m | 6.41 | 5.37 | 11.78 |
|  | 6 | 2.32 | 1.26 | 1.06 | 1.21 | 46 | – | m | 6.17 | 5.17 | 11.34 |
|  | 7 | 2.21 | 1.17 | 1.04 | 1.13 | 47 | – | m | 5.72 | 5.06 | 10.77 |
|  | 8 | 2.85 | 1.34 | 0.82 | 1.66 | 29 | 0.82 | sm | 6.56 | 8.01 | 13.93 |
| Bucak-2 | 1 | 3.52 | 2.18 | 1.34 | 1.65 | 38 | – | m | 8.69 | 5.32 | 14.01 |
|  | 2 | 3.4 | 1.87 | 1.53 | 1.23 | 45 | – | m | 7.46 | 6.1 | 13.56 |
|  | 3 | 3.24 | 1.75 | 1.49 | 1.18 | 46 | – | m | 6.97 | 5.94 | 12.91 |
|  | 4 | 3.11 | 1.81 | 1.29 | 1.41 | 42 | – | m | 7.22 | 5.15 | 12.38 |
|  | 5 | 2.97 | 1.58 | 1.39 | 1.15 | 47 | – | m | 6.28 | 5.53 | 11.81 |
|  | 6 | 2.74 | 1.48 | 1.26 | 1.19 | 46 | – | m | 5.9 | 5.0 | 10.9 |
|  | 7 | 2.49 | 1.4 | 1.09 | 1.31 | 44 | – | m | 5.57 | 4.33 | 9.9 |
|  | 8 | 3.65 | 1.6 | 1.02 | 1.59 | 28 | 1.03 | sm | 6.38 | 8.15 | 14.53 |
| Hüyük-2 | 1 | 3.76 | 2.24 | 1.51 | 1.48 | 41 | – | m | 8.45 | 5.72 | 14.17 |
|  | 2 | 3.49 | 1.93 | 1.56 | 1.26 | 45 | – | m | 7.27 | 5.91 | 13.17 |
|  | 3 | 3.4 | 2.18 | 1.22 | 1.8 | 36 | – | sm | 8.24 | 4.59 | 12.84 |
|  | 4 | 3.21 | 1.81 | 1.4 | 1.3 | 44 | – | m | 6.83 | 5.27 | 12.1 |
|  | 5 | 3.0 | 1.7 | 1.3 | 1.36 | 43 | – | m | 6.41 | 4.91 | 11.32 |
|  | 6 | 2.9 | 1.53 | 1.37 | 1.12 | 47 | – | m | 5.77 | 5.17 | 10.93 |
|  | 7 | 2.65 | 1.45 | 1.2 | 1.21 | 45 | – | m | 5.47 | 4.54 | 10.0 |
|  | 8 | 4.1 | 1.73 | 1.08 | 1.6 | 27 | 2.09 | sm | 6.55 | 11.93 | 15.49 |
| Akşehir-2 | 1 | 3.07 | 1.82 | 1.25 | 1.46 | 41 | – | m | 8.13 | 5.59 | 13.71 |
|  | 2 | 2.82 | 1.7 | 1.13 | 1.52 | 40 | – | m | 7.58 | 5.03 | 12.61 |
|  | 3 | 2.77 | 1.53 | 1.23 | 1.26 | 45 | – | m | 6.85 | 5.51 | 12.36 |
|  | 4 | 2.69 | 1.45 | 1.24 | 1.17 | 46 | – | m | 6.47 | 5.54 | 12.01 |
|  | 5 | 2.68 | 1.44 | 1.24 | 1.17 | 46 | – | m | 6.44 | 5.54 | 11.98 |
|  | 6 | 2.5 | 1.36 | 1.14 | 1.2 | 46 | – | m | 6.08 | 5.08 | 11.16 |
|  | 7 | 2.39 | 1.26 | 1.13 | 1.11 | 47 | – | m | 5.62 | 5.05 | 10.67 |
|  | 8 | 3.47 | 1.47 | 0.91 | 1.64 | 26 | 1.09 | sm | 6.56 | 8.93 | 15.5 |
| Uluborlu-1 | 1 | 4.64 | 2.71 | 1.94 | 1.41 | 42 | – | m | 8.3 | 5.93 | 14.24 |
|  | 2 | 4.31 | 2.72 | 1.59 | 1.73 | 37 | – | sm | 8.34 | 4.86 | 13.2 |
|  | 3 | 4.15 | 2.31 | 1.85 | 1.26 | 45 | – | m | 7.07 | 5.66 | 12.73 |
|  | 4 | 3.93 | 2.47 | 1.46 | 1.7 | 37 | – | sm | 7.57 | 4.49 | 12.06 |
|  | 5 | 3.84 | 2.04 | 1.8 | 1.14 | 47 | – | m | 6.27 | 5.52 | 11.79 |
|  | 6 | 3.61 | 1.88 | 1.73 | 1.08 | 48 | – | m | 5.75 | 5.31 | 11.06 |
|  | 7 | 3.45 | 1.8 | 1.65 | 1.09 | 48 | – | m | 5.52 | 5.06 | 10.58 |
|  | 8 | 4.67 | 2.05 | 1.3 | 1.63 | 28 | 1.32 | sm | 6.3 | 8.04 | 14.33 |
| Yalvaç-3 | 1 | 3.62 | 2.1 | 1.52 | 1.4 | 42 | – | m | 8.03 | 5.8 | 13.82 |
|  | 2 | 3.42 | 2.15 | 1.27 | 1.68 | 37 | – | sm | 8.19 | 4.86 | 13.05 |
|  | 3 | 3.36 | 1.88 | 1.48 | 1.27 | 44 | – | m | 7.17 | 5.66 | 12.82 |
|  | 4 | 3.16 | 1.82 | 1.34 | 1.37 | 42 | – | m | 6.94 | 5.11 | 12.05 |
|  | 5 | 3.11 | 1.69 | 1.41 | 1.21 | 45 | – | m | 6.46 | 5.38 | 11.85 |
|  | 6 | 2.93 | 1.56 | 1.37 | 1.15 | 47 | – | m | 5.93 | 5.23 | 11.16 |
|  | 7 | 2.62 | 1.38 | 1.25 | 1.11 | 48 | – | m | 5.25 | 4.76 | 10.01 |
|  | 8 | 3.99 | 1.99 | 0.99 | 2.02 | 25 | 1.02 | sm | 7.58 | 7.65 | 15.23 |
| Şuhut-2 | 1 | 3.47 | 1.96 | 1.5 | 1.32 | 43 | – | m | 7.63 | 5.85 | 13.48 |
|  | 2 | 3.31 | 2.08 | 1.23 | 1.69 | 37 | – | sm | 8.09 | 4.79 | 12.88 |
|  | 3 | 3.2 | 1.88 | 1.32 | 1.44 | 41 | – | m | 7.32 | 5.13 | 12.45 |
|  | 4 | 3.11 | 1.7 | 1.41 | 1.21 | 45 | – | m | 6.6 | 5.48 | 12.08 |
|  | 5 | 3.02 | 1.65 | 1.37 | 1.21 | 45 | – | m | 6.41 | 5.33 | 11.74 |
|  | 6 | 2.93 | 1.61 | 1.32 | 1.22 | 45 | – | m | 6.24 | 5.14 | 11.38 |
|  | 7 | 2.72 | 1.43 | 1.29 | 1.11 | 47 | – | m | 5.55 | 5.02 | 10.58 |
|  | 8 | 3.96 | 1.69 | 0.92 | 1.85 | 23 | 1.36 | sm | 6.57 | 8.85 | 15.41 |
| Bolvadin-3 | 1 | 3.43 | 2.06 | 1.37 | 1.51 | 40 | – | m | 8.58 | 5.71 | 14.29 |
|  | 2 | 3.16 | 1.79 | 1.37 | 1.32 | 43 | – | m | 7.47 | 5.69 | 13.17 |
|  | 3 | 3.03 | 1.75 | 1.28 | 1.38 | 42 | – | m | 7.28 | 5.32 | 12.61 |
|  | 4 | 2.91 | 1.58 | 1.33 | 1.19 | 46 | – | m | 6.58 | 5.55 | 12.13 |
|  | 5 | 2.75 | 1.49 | 1.26 | 1.19 | 46 | – | m | 6.2 | 5.25 | 11.45 |
|  | 6 | 2.63 | 1.35 | 1.29 | 1.05 | 49 | – | m | 5.61 | 5.36 | 10.97 |
|  | 7 | 2.43 | 1.27 | 1.16 | 1.1 | 48 | – | m | 5.3 | 4.82 | 10.13 |
|  | 8 | 3.66 | 1.46 | 0.93 | 1.6 | 25 | 1.19 | sm | 6.1 | 8.84 | 15.26 |
